# Turning over New Leaves

**Published:** 1889-06-01

**Authors:** 


### Turning Over New Leaves.

(Books for Review should be sent to The Editor, The Lodge, Porchester Square, W.)

### THE CAUSE OF CHOLERA.*

Dr. Klein's book on cholera, though it appears among Macmillan's "Manuals for Students," is of too controversial a character to be rightly included among them. Originally published as a series of articles in the *Practitioner*, it is chiefly occupied with a contradiction of Koch's views as to the comma bacillus of cholera, and a protest against Koch's misinterpretation of Dr. Klein's views. Both scientists agree that the comma bacillus is found in the dejecta of cholera patients, and in the intestines of those who have died from the disease, particularly in the ileum, and the lower part of the duodenum. But Dr. Klein has not found them in such numbers as Koch, nor with such regularity as to justify him in regarding them as a cause rather than a symptom of the disease. The true bacteria of Asiatic cholera are, he considers, yet to be found, and certainly his researches leave the matter vague. For sanitarians and the bulk of the medical profession, the question is interesting only in so far as it may help the prevention of the disease. If the comma bacillus were proved to be the distinctive cause of infection in Asiatic cholera, preventive measures could be confined to the deprivation of a suitable soil for its development; but while specialists differ so much all efforts must be more or less speculative and

* "The Bacteria in Asiatic Cholera." By E. Klein, M.D., F.R.S., Professor of Bacteriology at the College of State Medicine, London, etc. 1s. London: Macmillan and Co.

empirical, and the common rules of hygiene are the best that can be followed.

### THE NUN OF KENMARE.*

This being a controversial volume on questions pertaining to the treatment of a nun in the Catholic Church by Catholic prelates and others of authority, we do not deem it suitable for an exhaustive review in these columns; but being sent to us by the publishers, we feel bound to notice it briefly. It is really the autobiography of a convert from the Church of England to that of Rome, who, like so many others, did not find the change wholly satisfactory in some respects, but unlike them, now publishes her feelings on the housetops. A considerable part of the volume treats of the Catholic Church in America. This is one questionable statement which is made concerning it: "It must be admitted, however, that no other religious body is so dependent upon the liquor saloon interest as the Roman Catholic Church. In fact, it is the only religious body that looks to this interest for its support; and it is but justice to say that if the money obtained from this source, directly as well as indirectly, were withdrawn, some institutions would have a poor look out. The liquor saloon keeper who bosses the wards knows how to obtain Government money and subsidies for orphan and other institutions, and with the most free and generous hand these men contribute to every Roman Catholic charity."

* "The Nun of Kenmare." Hodder and Stoughton.

